# Supplementary material for: Visualising G-quadruplex DNA dynamics in live cells by fluorescence lifetime imaging microscopy
Source: Nat Commun. 2021 Jan 8;12:162. doi: 10.1038/s41467-020-20414-7 (PMC7794231; doi:10.1038/s41467-020-20414-7)
Supplement: Supplementary file 2 — Reporting Summary [file 41467_2020_20414_MOESM2_ESM.pdf]

nature research

Corresponding author(s): Ramon Vilar

Last updated by author(s): Nov 20, 2020

Reporting Summary

Nature Research wishes to improve the reproducibility of the work that we publish. This form provides structure for consistency and transparency in reporting. For further information on Nature Research policies, seeAuthors & Referees and theEditorial Policy Checklist .

Statistics

For all statistical analyses, confirm that the following items are present in the figure legend, table legend, main text, or Methods section.

n/a	Confirmed	
☐	☒	The exact sample size (*n*) for each experimental group/condition, given as a discrete number and unit of measurement
☐	☒	A statement on whether measurements were taken from distinct samples or whether the same sample was measured repeatedly
☐	☒	The statistical test(s) used AND whether they are one- or two-sided<br>*Only common tests should be described solely by name; describe more complex techniques in the Methods section.*
☒	☐	A description of all covariates tested
☒	☐	A description of any assumptions or corrections, such as tests of normality and adjustment for multiple comparisons
☐	☒	A full description of the statistical parameters including central tendency (e.g. means) or other basic estimates (e.g. regression coefficient) AND variation (e.g. standard deviation) or associated estimates of uncertainty (e.g. confidence intervals)
☐	☒	For null hypothesis testing, the test statistic (e.g. *F*, *t*, *r*) with confidence intervals, effect sizes, degrees of freedom and *P* value noted<br>*Give P values as exact values whenever suitable.*
☒	☐	For Bayesian analysis, information on the choice of priors and Markov chain Monte Carlo settings
☒	☐	For hierarchical and complex designs, identification of the appropriate level for tests and full reporting of outcomes
☒	☐	Estimates of effect sizes (e.g. Cohen's *d*, Pearson's *r*), indicating how they were calculated

Our web collection on statistics for biologists contains articles on many of the points above.

Software and code

Policy information about availability of computer code

Data collection	Fluorescence Lifetime Imaging Microscopy data collection: SPCM v9.83 (Becker & Hickl GmbH).<br>Time Correlated Single Photon Counting decay collection: DataStation v2.2 (HORIBA Scientific).<br>Confocal fluorescence image collection: Las AF v2.6.0 (Leica).<br>Carl Zeiss software to take images on microscope for gammaH2AX staining: ZEN 2 version number: 2.0.14283.302<br>Flowjo version number: 10.7
Data analysis	Fluorescence Lifetime Imaging Microscopy data fitting: FLIMfit v5.1.1 (Sean Warren, Imperial College London).<br>Time Correlated Single Photon Counting decay fitting: DAS6 v6.8 (HORIBA Scientific).<br>Confocal fluorescence image processing: Las AF v2.6.0 (Leica).<br>Statistical analysis: OriginPro 9.55 (OriginLab).

For manuscripts utilizing custom algorithms or software that are central to the research but not yet described in published literature, software must be made available to editors/reviewers. We strongly encourage code deposition in a community repository (e.g. GitHub). See the Nature Research guidelines for submitting code & software for further information.

Data

Policy information about availability of data

All manuscripts must include a data availability statement. This statement should provide the following information, where applicable:

- Accession codes, unique identifiers, or web links for publicly available datasets
- A list of figures that have associated raw data
- A description of any restrictions on data availability

Data that support the findings of this study have been deposited in Biorxiv. Source data for all Figures (where applicable) are provided with an example raw FLIM

data file (used in Figure 2). Web links and DOI numbers for the source data on Biorxiv will be made available before final publication.

Field-specific reporting

Please select the one below that is the best fit for your research. If you are not sure, read the appropriate sections before making your selection.

☒ Life sciences ☐ Behavioural & social sciences ☐ Ecological, evolutionary & environmental sciences

For a reference copy of the document with all sections, see nature.com/documents/nr-reporting-summary-flat.pdf

Life sciences study design

All studies must disclose on these points even when the disclosure is negative.

Sample size	No sample-size calculation was performed, as this study did not include animal models or human participants. Sample sizes for each experiment were determined from a sample size required to give a reproducible average, and high statistical significance, based on standards in the field (Demeautis, C. et al. Sci. Rep. 7, 41026; doi: 10.1038/srep41026 (2017)). For example, in a typical FLIM experiment, three images were taken (each image containing approximately 10-30 cells) for each condition. This was repeated on a biologically independent sample (independent cell batch). The results of the two repeats were pooled, and statistics performed.
Data exclusions	For FLIM data, cell nuclei that did not meet threshold conditions (intensity at decay maximum, or Chi2) described in the methods section were not included in the statistical analysis. These conditions were determined to exclude low-intensity nuclei where the lifetime was too noisy to be accurately fitted by the biexponential model (Becker, W. "The bh TCSPC Handbook", 8th edition (2019)). Additionally, it was predetermined that cells which had unusual morphology were not included in the statistical analysis to exclude the influence of cells entering apoptosis on the data analysis. These parameters were applied consistently to all imaging studies.
Replication	All experimental findings in the manuscript were reliably reproduced as indicated in the figure legends.
Randomization	Randomization is not applicable to this type of study because this study did not involve animals or human participants. Samples were organized into groups based on treatment and genotype. Appropriate controls were included in all experiments.
Blinding	There was no blinded group allocation because of the nature of biological samples and type of experiments performed. Data sets were reprocessed independently by 2 different researchers to validate the results.

Reporting for specific materials, systems and methods

We require information from authors about some types of materials, experimental systems and methods used in many studies. Here, indicate whether each material, system or method listed is relevant to your study. If you are not sure if a list item applies to your research, read the appropriate section before selecting a response.

Materials & experimental systems

n/a	Involved in the study	
☐	☒	Antibodies
☐	☒	Eukaryotic cell lines
☒	☐	Palaeontology
☒	☐	Animals and other organisms
☒	☐	Human research participants
☒	☐	Clinical data

Methods

n/a	Involved in the study	
☒	☐	ChIP-seq
☐	☒	Flow cytometry
☒	☐	MRI-based neuroimaging

Antibodies

Antibodies used	Antibodies used for immunofluorescence and Western blotting:<br>Beta-actin, Mouse, Abcam ab8226<br>BRIP1/FANCJ, Rabbit, Novus NB1-31883<br>Anti-mouse Ig-HRP, Invitrogen A16078<br>Anti-rabbit Ig-HRP, Dako P0217<br>gH2AX, Millipore 05-636<br>Anti-mouse Alexa 488, Life Technologies A11001<br>Anti-rabbit RTEL1, Novus Biologicals NBP2-22360
Validation	Primary antibodies are used according to the commercial suppliers protocols (Abcam, Novus, Introgen, Dako, Millipore, and Life Technologies) and validated against strains that do not expressed relevant epitopes:

Beta-actin, Mouse, Abcam ab8226 (confirmed on the Abcam website as suitable for: ICC/IF, ICC, Flow Cyt, IHC-FrFl, IHC-P, IHC-Fr, IP, WB). Validated in: Rap1-GTPases control mTORC1 activity by coordinating lysosome organization with amino acid availability. Nat Commun. 2020 Mar 17;11(1):1416. doi: 10.1038/s41467-020-15156-5. PMID: 32184389

BRIP1/FANCJ, Rabbit, Novus NB1-31883 (confirmed on the Novus website as suitable for: WB, ICC/IF, IHC, IHC-P). Validated in: FANCJ suppresses microsatellite instability and lymphomagenesis independent of the Fanconi anemia pathway. Genes Dev. 2015 Dec 15; 29(24): 2532–2546. doi: 10.1101/gad.272740.115. PMID: 26637282

gH2AX, Mouse, Millipore 05-636 (confirmed on the Milipore website as suitable for: ICC, IF, WB, ChIP, IHC). Validated in: The apoptotic ring: A novel entity with phosphorylated histones H2AX and H2B, and activated DNA damage response kinases. Cell Cycle. 2009, 8:12, 1853-1859, DOI: 10.4161/cc.8.12.8865

Anti-rabbit RTEL1, Novus Biologicals NBP2-22360 (conformed on the Novus website as suitable for: WB). Validated in: Stabilization of Reversed Replication Forks by Telomerase Drives Telomere Catastrophe. Cell. 2018 Jan 25; 172(3): 439–453.e14. doi: 10.1016/j.cell.2017.11.047. PMID: 29290468

Secondaries:
Anti-mouse Ig-HRP, Invitrogen A16078 (confirmed on the Invitrogen website as suitable for: ELISA, IHC, WB)
Anti-rabbit Ig-HRP, Dako P0217 (confirmed on the Dako website as suitable for: ICC, ELISA, WB)

Eukaryotic cell lines

Policy information about cell lines

Cell line source(s)	U2OS (ATCC)<br>MEF RTel1 F/F FancJ +/- (gift from S. Boulton, validated in Matsusaki et al., 2015)<br>MEF RTel1 F/F FancJ -/- (gift from S. Boulton, validated in Matsusaki et al., 2015)
Authentication	Good lab practice recommended by ATCC was used to set a basic benchmark of cell verification: using low-passage cell lines, morphology checks, mycoplasma detection. MEF cells were deposited at the Crick Institute Cell services after validation in Matsusaki et al., 2015.
Mycoplasma contamination	We confirm that all our cell lines used in this manuscript have been tested negative for mycoplasma contamination.
Commonly misidentified lines (See ICLAC register)	No cell lines appear on the Register of Misidentified Cell Lines

Flow Cytometry

Plots

Confirm that:

- [x] The axis labels state the marker and fluorochrome used (e.g. CD4-FITC).
- [x] The axis scales are clearly visible. Include numbers along axes only for bottom left plot of group (a 'group' is an analysis of identical markers).
- [x] All plots are contour plots with outliers or pseudocolor plots.
- [x] A numerical value for number of cells or percentage (with statistics) is provided.

Methodology

Sample preparation	For measurement of FITC fluorescence intensity, 5x10^5 cells were resuspended in FACS buffer (PBS containing Propidium Iodide) and analyzed. For cell cycle analysis, cells were incubated with BrdU (10µM), harvested, stained with αBrdU-FITC conjugate (BD), and then with PI (10µg/ml) before analysis.
Instrument	BD FACS Aria Fusion
Software	Data Collection: BD FACS Diva (v8.0.1)<br>Data analysis: FlowJo software (v10.7.1)
Cell population abundance	For MEFs, an average of 50,000 cells were counted for each condition. For U2OS, 15,000 and more cells were analysed. See raw data file.
Gating strategy	FSC and SSC gates were set for doublet exclusion. See raw data file.

- [x] Tick this box to confirm that a figure exemplifying the gating strategy is provided in the Supplementary Information.
